# Spatial Distribution and Long-Term Persistence of *Wolbachia*-Infected *Aedes aegypti* in the Mentari Court, Malaysia

**DOI:** 10.3390/insects14040373

**Published:** 2023-04-11

**Authors:** Yoon Ling Cheong, Wasi A. Nazni, Han Lim Lee, Ahmad NoorAfizah, Ibrahim C. MohdKhairuddin, Ghazali M. R. Kamarul, Nasir M. N. Nizam, Mohd A. K. Arif, Zabari M. NurZatilAqmar, Saidin M. Irwan, Khairuddin Khadijah, Yusof M. Paid, Omar Topek, Asim H. Hasnor, Rahman AbuBakar, Balvinder Singh Gill, Kamaludin Fadzilah, Aris Tahir, Steven P. Sinkins, Ary A. Hoffmann

**Affiliations:** 1 Institute for Medical Research, National Institutes of Health, Ministry of Health Malaysia, Jalan Pahang, Kuala Lumpur 50588, Wilayah Persekutuan Kuala Lumpur, Malaysia; 2 Petaling District Health Office, Ministry of Health Malaysia, SS 6, Petaling Jaya 47301, Selangor, Malaysia; 3 Federal Territory Health Department of Kuala Lumpur and Putrajaya, Ministry of Health Malaysia, Jalan Cenderasari, Tasik Perdana, Kuala Lumpur 50480, Wilayah Persekutuan Kuala Lumpur, Malaysia; 4 Institute for Health Behavioural Research, National Institutes of Health, Ministry of Health Malaysia, Jalan Setia Murni U13/52, Seksyen U13 Setia Alam, Shah Alam 40170, Selangor, Malaysia; 5 MRC—University of Glasgow Centre for Virus Research, 464 Bearsden Road, Glasgow G61 1QH, UK; 6 Bio21 Institute and the School of BioSciences, University of Melbourne, 30 Flemington Road, Parkville, VIC 3052, Australia

**Keywords:** spatial interpolation, *Ae. aegypti*, *Wolbachia*, ovitrap index, invasion

## Abstract

**Simple Summary:**

The mosquito *Aedes aegypti* is the primary vector of the dengue virus in humans. *Wolbachia* bacteria can prevent dengue virus transmission following transfer into this species. *Ae. aegypti* of both sexes carrying *Wolbachia* strain *w*AlbB were released in Mentari Court, Malaysia, in October 2017 for 20 weeks. This study aims to investigate the spatial distribution of *Wolbachia*-carrying mosquitoes in a high-rise residential site and examine the nature of the composition of the mosquito population across the site. There are numerous findings; firstly, *Wolbachia*-infected mosquitoes showed a high infection frequency four years after release. Secondly, there were some minor effects of floors and blocks on the frequency of *Wolbachia*. Thirdly, there was no evidence that the *Aedes albopictus* increased across the area. While the invasion of *Wolbachia* will vary from location to location, this study filled a knowledge gap on the invasion of such a strain in a high-rise residential area. This study can assist in planning field release strategies and the development of models that can forecast local success.

**Abstract:**

Dengue is endemic in Malaysia, and vector control strategies are vital to reduce dengue transmission. The *Wolbachia* strain *w*AlbB carried by both sexes of *Ae. aegypti* was released in Mentari Court, a high-rise residential site, in October 2017 and stopped after 20 weeks. *Wolbachia* frequencies are still being monitored at multiple traps across this site, providing an opportunity to examine the spatiotemporal distribution of *Wolbachia* and mosquito density with respect to year, residential block, and floor, using spatial interpolation in ArcGIS, GLMs, and contingency analyses. In just 12 weeks, *Wolbachia*-infected mosquitoes were established right across the Mentari Court site with an overall infection frequency of >90%. To date, the *Wolbachia* frequency of *Ae. aegypti* has remained high in all areas across the site despite releases finishing four years ago. Nevertheless, the *Wolbachia* invaded more rapidly in some residential blocks than others, and also showed a relatively higher frequency on the eighth floor. The *Ae. aegypti* index tended to differ somewhat between residential blocks, whilst the *Ae. albopictus* index was relatively higher at the top and bottom floors of buildings. In Mentari Court, only a short release period was required to infiltrate *Wolbachia* completely and stably into the natural population. The results inform future releases in comparable sites in a dengue control programme.

## 1. Introduction

Dengue is estimated to infect more than 390 million people annually in tropical and subtropical regions worldwide [1]. Another study [2] on the prevalence of dengue estimates that 3.97 billion people are at risk of infection with dengue viruses. Despite a risk of infection existing in 129 countries [2], 70% of the actual burden is in Asia [1]. While dengue vaccine development is ongoing, made more challenging by complex patterns of immunity against the four dengue serotypes, vector control interventions are used to combat dengue transmission [2,3]. Insecticide spraying, mosquito trap deployment, mass insecticide fogging, and other methods are used to temporarily stop the spread of dengue; however, these methods are not sustainable. The over usage of insecticides has resulted in increased levels of insecticide resistance in the vector species [4,5]. Hence, new biological control methods are needed, and the release of *Wolbachia*-infected mosquitoes provides one of the new vector control strategies to control the spread of arboviral diseases including dengue, Zika, and chikungunya.

Field releases of *Wolbachia* strains in several tropical/sub-tropical countries have been conducted using two strategies: suppression and replacement. Suppression, also known as the Incompatible Insect Technique (IIT) [6], aims to release only *Wolbachia*-carrying males into a vector population uninfected by *Wolbachia*, which can reduce vector numbers, particularly in isolated areas, and has been implemented in Singapore [7], China [8], Australia [9], and the United States [10]. On the other hand, replacement, a strategy of releasing both male and female *Wolbachia*-infected mosquitoes, aims to replace a local population of *Wolbachia*-free mosquitoes (or a population carrying a different strain of *Wolbachia*) and has been implemented in Australia [11,12], Brazil [13], Indonesia [14], Vietnam [15], and Malaysia [16]. While the suppression strategy is intended to decrease host population density, the replacement strategy focuses on reducing host–virus transmission [17]. Although promising, the success of both strategies can be challenged by various environmental conditions, virus-blocking levels, and fitness costs of variants [17]. Mathematical models have been used to estimate the spatial spread of a *Wolbachia* transinfection based on varying release frequency, strategy, and area [18]. Models of *Wolbachia* invasion can be improved based on empirical data from field investigations [19,20].

In Malaysia, Nazni et al. [16] reported the release of the *Ae. aegypti w*AlbB *Wolbachia* strain in more than six locations with the successful stable invasion of several field populations since 2017. One of the first sites where releases were undertaken involved Mentari Court, Selangor, where a total of 40,800 *Wolbachia*-infected adult *Ae. aegypti* (both sexes) were released weekly for four and a half months, from 16 October 2017 to 5 March 2018, with the first monitoring conducted four weeks after the release [16]. The *Wolbachia*-infected mosquitoes were released at the residential area, car park, and outdoor area within external fences, covering the ground, 2nd, 5th, 8th, 11th, 14th, and 17th floor of residential Blocks A to F, and the 1st and 3rd floors of the car park buildings. The frequency of *Wolbachia* increased to above 90% after eleven weeks of adult release and remained stably high two years afterward [21]. According to Nazni et al. and Lau et al. [16,22] this location was known as a dengue hotspot locality with a dengue incidence of 7371 per 100,000 population from January 2013 until the start of the intervention. Numerous other control interventions have been used at this site, but all failed to stop local dengue transmission. However, no significant dengue outbreaks have been documented since the release of *Wolbachia*-infected mosquitoes in this area. The estimated 40% decrease in dengue cases across all project study sites in comparison to control sites supports the efficacy of the *Wolbachia* strategy [16].

Studies in Cairns, Australia [12] and Yogyakarta, Indonesia [14,23] have described the spatial dispersal of the *Wolbachia* strain *w*Mel *Ae. aegypti* in low-rise house areas following release. However, Malaysia is the first country to implement replacement through releases of *Wolbachia w*AlbB *Ae. aegypti* in high-rise residential buildings and we, therefore, used this opportunity to track the persistence and invasion of *Wolbachia* at a spatial level within this context. Previous genomic studies at Mentari Court based on kinship relatedness have highlighted movement patterns of adult mosquitoes in this environment, which tend to occur mostly within buildings in the same generation but with sporadic movement across buildings when multiple generations are considered [24], suggesting that released *Ae. aegypti* will move within residential buildings at this location [25]. Therefore, the objectives of this study are to (i) test the spatial distribution of *Wolbachia*-induced mosquitoes in a high-rise residential site, and (ii) examine the nature of the composition of the mosquito population across the site.

## 2. Materials and Methods

### 2.1. Study Site

**Figure 1:**
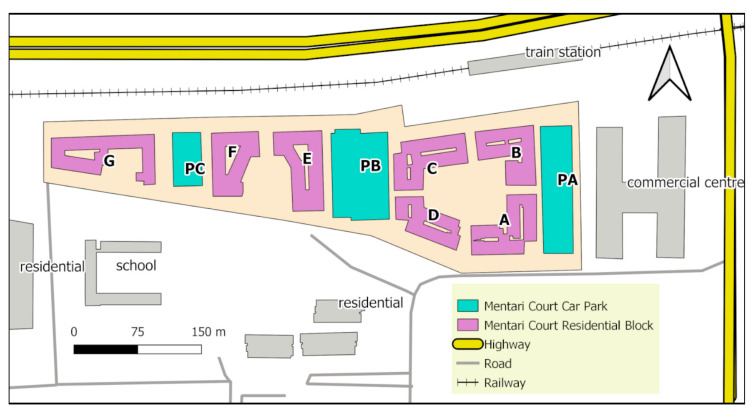
Map of Mentari Court; Block A, B, C, D, E, F & G are residential blocks; Block PA, PB and PC are car parks blocks.

Mentari Court (3°04′55.2″ N, 101°36′39.3″ E) is a low-cost community housing apartment that consists of seven eighteen-floor residential blocks and three car park blocks with 3472 units [16] (Figure 1). Several other residential blocks and a school surround this area at a distance of around 100 m from Mentari Court, with these areas separated by shrubs and an open space car park. To the north, a six-lane highway, known as the “Federal Highway”, is more than 100 m away from the nearest release point. The total build-up of the area covers 90,267 m^2^, with two three-floor car parks between the blocks (Figure 1).

### 2.2. Data Collection

The first ovitrap monitoring in Mentari Court was conducted four weeks after release, and this was continued every two weeks for seven months. Subsequently, monitoring was conducted once a month for the next six months, once every two months in 2019 and 2020, once every five months from February to June 2020, and once every six months since April 2021. For each monitoring event, 100 ovitraps, each with 150 mL water and a wooden ovipositor, were set in designated sites and collected after one week [16]. A maximum of 10 *Ae. aegypti* mosquitoes were haphazardly selected from each positive trap for a PCR test of *Wolbachia* frequency and density [16]. The frequency of *Wolbachia* was calculated based on the percentage of positive *Wolbachia*-infected *Ae. aegypti* over the minimum of one tested *Ae. aegypti*. Overall, *Wolbachia* frequencies, the *Ae. aegypti* index, and the *Ae. albopictus* index before 15 January 2019 was reported by Nazni et al. [16]. The data were aggregated by block and floor. The dengue-confirmed cases were provided by the e-Dengue vector surveillance system of the Vector Control Department, Ministry of Health Malaysia. The data were recorded in a MySQL database.

### 2.3. Geospatial Analysis

Only trapping data, where at least four *Ae. aegypti* were found, were included in the analysis. The building technical drawing map of Mentari Court was supplied by the Joint-Management Board (JMB) and was then georeferenced and translated into a digital shapefile map of the study site (Figure 1) using QGIS software. Interpolation analysis was performed with ordinary kriging using the ArcGIS Geostatistical Wizard for each of the 6 ovitrap monitoring periods (1st through 5th and 34th).

### 2.4. Statistical Analysis

We conducted a statistical analysis using IBM SPSS Statistics 22 on data pooled across traps placed on a particular floor on a sampling occasion. We analysed the *Wolbachia* frequency, the *Ae. aegypti* index (positive traps), and *Ae. albopictus* index (positive traps). For the *Wolbachia* frequencies of *Ae. aegypti*, we performed the Generalized Linear Model (GLM) with a binomial distribution and logit link function on the proportion of the total mosquito tested with positive *Wolbachia* over the total mosquitos tested. We compared the *Wolbachia* frequencies across the years, blocks, and floors. We included only data after 2017 beyond the release period, the total number of tested mosquitoes more than zero, and the residential block with 17 floors. Due to the different structures of the residential blocks in comparison to car parks, we focused the analysis on the residential blocks. Rstudio version 1.2.1335 and packages ggplot2, dplyr, lubridate, and tidyr were used for the chart plots.

The analysis of the *Ae. aegypti* index was similar to the analyses undertaken on the *Wolbachia* frequencies. We used a GLM with a binomial distribution and logit link function to analyze the proportion of positive traps with *Ae. aegypti* in comparison to the number of recovered traps, considering the different factors that might influence this proportion. The analysis of *Ae. albopictus* index values was kept much simpler due to the low number of traps positive for this species. Here, contingency tests were applied to examine the effects of blocks and floors on the presence of this species. We used a G-test and assessed the significance using a Monte Carlo procedure. We also combined data from the middle floors in a further contingency test to compare the incidence of *Ae. albopictus* in the middle versus the top and ground floors.

## 3. Results

**Figure 2:**
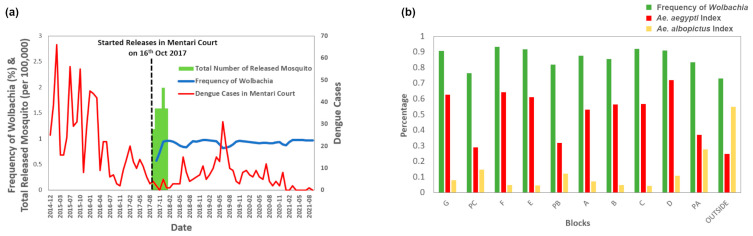
(**a**) Frequency of *Wolbachia*, dengue cases in Mentari Court before and after release and total released mosquitoes, and (**b**) average frequency of *Wolbachia*, the *Ae. aegypti* index, and the *Ae. albopictus* index in Mentari Court by blocks from 16 October 2017 to 5 October 2021.

The prevalence of *Wolbachia* in Mentari Court is relatively steady with *Wolbachia*-infected mosquitoes at over 80% even after four years since releases started in 2017 (Figure 2a). Dengue cases showed a declining trend since the release of *Wolbachia*-infected mosquitoes (Figure 2a). In all blocks, *Wolbachia* abundance was generally lower in the car parks, similar to the trend in the *Ae. aegypti* index (Figure 2b). In contrast, the *Ae. albopictus* index showed the opposite pattern, being higher in the car parks and outside areas near the Mentari Court fences compared to inside the residential blocks (Figure 2b).

**Figure 3:**
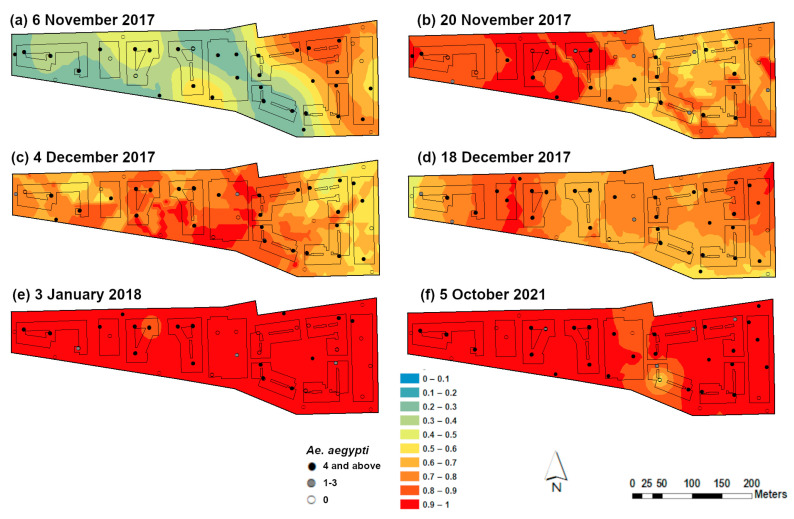
Spatial distribution of *Wolbachia*-infected mosquitoes in Mentari Court based on ordinary kriging. The analysis is only based on the ovitraps with four or more *Ae. aegypti*. Red colour indicates a higher frequency of *Wolbachia*-infected mosquitoes.

Interpolation analysis of frequency data in the period during and shortly after releases were conducted [16] showed that after the first three weeks of release, the highest *Wolbachia* frequencies were in block B and part of blocks A and C (Figure 3a). After two weeks, the *Wolbachia* had spread to the other side of the locality (Figure 3b). In the subsequent two weeks, the third monitoring period, there was a higher abundance of *Wolbachia* in the centre of the study area, i.e., blocks C, D, E, and F (Figure 3c). Fluctuating *Wolbachia* frequencies were still observed two months after the initial release (Figure 3d). Nevertheless, about 12 weeks after the initial release (on 3 January 2018), all blocks of Mentari Court were successfully invaded by *Wolbachia* (Figure 3e). After four years of release, the *Wolbachia*-infected mosquitoes showed stably high frequencies (>80%) across Mentari Court (Figure 3f).

**Figure 4:**
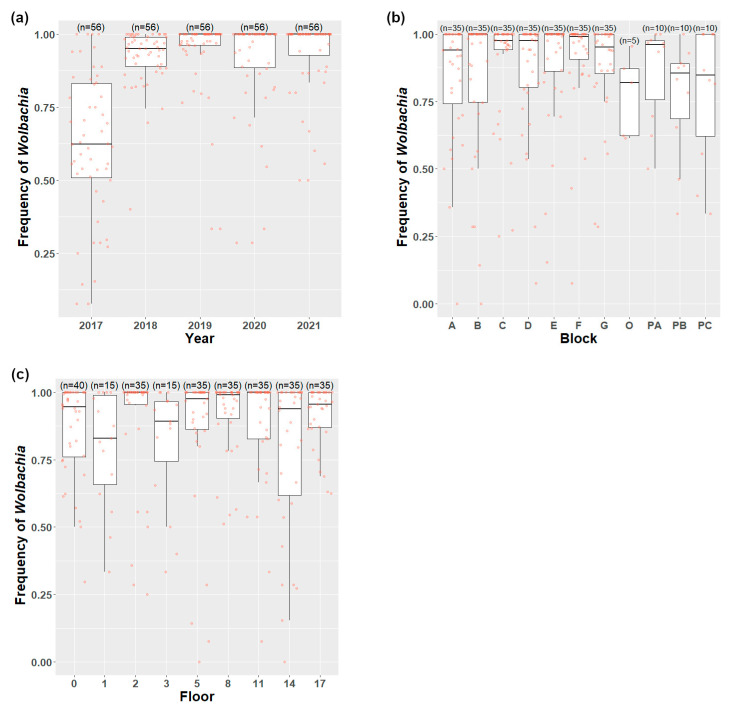
*Wolbachia* frequencies of *Ae. aegypti* strain *w*AlbB by (**a**) year, (**b**) blocks, and (**c**) floors in Mentari Court (O is the abbreviation for the outside area).

The *Wolbachia* frequencies of *Ae. aegypti* were stable across the years following the invasion (Figure 4a), and those in the three car parks and outside areas were generally lower than in the residential blocks (Figure 4b). The analysis of the residential blocks indicates that the *Wolbachia* frequencies of *Ae. aegypti* stayed stably high after release (*χ*^2^ = 11.28; d.f. = 3; *p* = 0.010) (Figure 4b). *Wolbachia* frequencies did not differ significantly across the residential blocks (*χ*^2^ = 7.82; d.f. = 6; *p* = 0.252). There was a significant difference between the *Wolbachia* frequencies of *Ae. aegypti* across the floors (*χ*^2^ = 15.83; d.f. = 5; *p* = 0.007). The *Wolbachia* frequencies on the 2nd and 8th floors were higher than on the 14th floor (Figure 4c).

**Figure 5:**
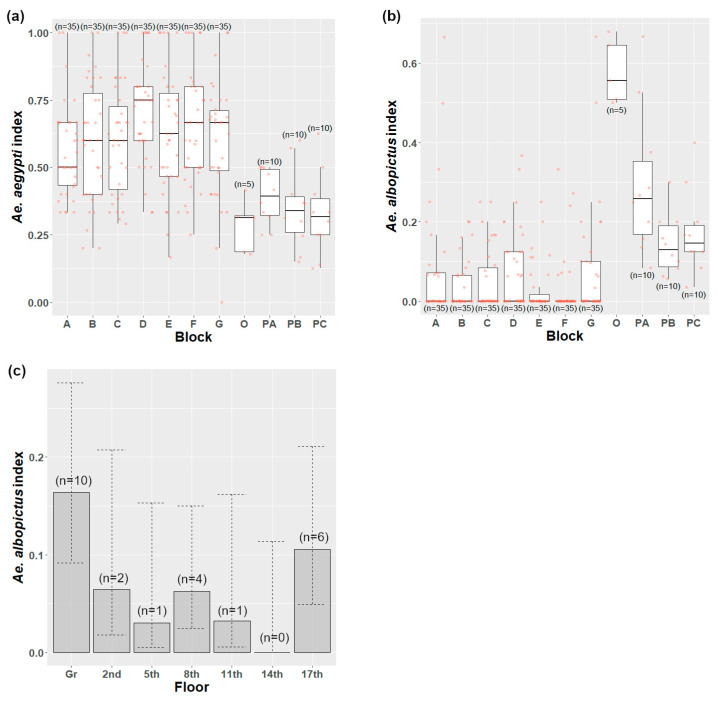
(**a**) *Ae. aegypti* index by blocks; (**b**) *Ae. albopictus* index by blocks; (**c**) *Ae. albopictus* index by floors in Mentari Court from 17 October 2017 to 2021.

The *Ae. aegypti* index was similar across the years since release (*χ*^2^ = 3.81; d.f. = 4; *p* = 0.433) and floors (*χ*^2^ = 5.95; d.f. = 6; *p* = 0.429). For the carparks and the outside area, the *Ae. aegypti* index was generally two-fold lower than for the residential blocks (Figure 5a). Across the residential blocks, the *Ae. aegypti* index showed a weak difference across the blocks (*χ*^2^ = 11.46; d.f. = 6; *p* = 0.075), with the *Ae. aegypti* index being the lowest in block A and the highest in block D.

The *Ae. albopictus* index was higher in the carparks and outside area than in the residential blocks (Figure 4b). A contingency test on pooled data indicated that there was a marginally non-significant difference among floors (from ground to 17th) (*χ*^2^ = 13.071; d.f. = 6; *p* = 0.065). There was no difference between the 2nd floor to 14th floor (*χ*^2^ = 3.699; d.f. = 4; *p* = 0.621). When the middle floors were combined, there was a significant difference in the proportion of positive traps among the top floors, bottom floors, and the combined middle floors (*χ*^2^ = 9.372; d.f. = 2; *p* = 0.015). Hence, across the residential blocks, the *Ae. albopictus* index tended to be highest at the top and bottom floors (Figure 5c).

## 4. Discussion

The results show that *Wolbachia* has successfully been established in Mentari Court for four years since 2017, after only 20 weeks of release. This study shows, spatially, the process of the establishment of *Wolbachia* in *Ae. aegypti* in high-rise residential apartments where wild populations were reported as being abundant [22], with a frequency of *Wolbachia* >90% being maintained. This period is shorter than the invasion period shown by the *w*Mel *Wolbachia* infection in some residential areas with single-level houses in Australia [26] and Indonesia [14]. Enclosed high-rise buildings may provide an ideal environment for invasion assuming that there is less movement of mosquitoes from external areas into the release zone. Since the release of *Wolbachia*-infected mosquitoes, there have been no major dengue outbreaks in Mentari Court, and the dengue cases have declined sharply. In addition, no mass thermal fogging activities have been carried out since the release, unlike in other areas where only source reduction of the vector population and a dengue awareness program have been undertaken [16].

Our detailed study of one site provides information on the possible interaction of *Wolbachia*-infected *Ae. aegypti* with wild *Ae. albopictus*. The *Wolbachia* invasion was generally slower in the parking blocks than in the residential blocks, and the *Ae. aegypti* numbers were lower in the parking blocks, which may be linked to more *Ae. albopictus* being present. Across the residential blocks, *Wolbachia* frequencies were higher on the second and eighth floor (the middle floor) than on other floors, whereas *Ae. albopictus* were more common on the top and ground floors and *Ae. aegypti* numbers did not differ across floors. Indirect competition between *Wolbachia*-infected *Ae. aegypti* and the *Ae. albopictus* for space was inferred by Tantowijoyo et al. [27] in Yogyakarta, Indonesia, where areas with *Ae. aegypti* that had a higher frequency of *Wolbachia* infection also had a lower density of *Ae. albopictus* and vice versa. There is potential niche competition between species for oviposition sites [28], and satyrisation can also affect interactions among these species [29]. However, at Mentari Court, the potential for competition is limited due to the low abundance of *Ae. albopictus*. We found that only 6% of traps had both species, whereas the percentage of traps with only *Ae. aegypti* or *Ae. albopictus* was 47% and 12%, respectively. The 6% figure fits well with the expected incidence of traps with both species if they are independent (9.5%). Note that we also observed no significant changes in the relative abundance of *Ae. aegypti* and *Ae. albopictus* after the *Wolbachia* invasion in *Ae. aegypti* across the four years, In line with an Indonesian study [30] and inconsistent with direct competition between these species. We, therefore, suspect that other factors correlated with *Ae. albopictus* influence *Wolbachia* invasion.

*Wolbachia* invasion rates within an area where there is restricted mosquito immigration from outside areas are expected to depend on the relative density of infected mosquitoes compared to uninfected mosquitoes. The *Wolbachia* invasion occurred faster in blocks A, B, and C during the first four weeks after release, which may be related to the higher densities of uninfected mosquitoes there [22]. This is in contrast with invasion rates associated with the spatial distribution of mosquitoes in Gordonvale, Australia [31] and Vietnam [15] which showed that residential blocks with a higher density of uninfected mosquitoes were less susceptible to *Wolbachia* invasion. Block G showed a slower invasion rate than the other blocks which could be related to the unique extended oblong shape of the buildings in that block, perhaps making these more prone to the introduction of uninfected mosquitoes from outside the area. While *Ae. aegypti* mosquitoes in Mentari Court did not spread beyond 25 m in the same generation and tended to be restricted to the same building [24], uninfected mosquitoes could have moved from outside the release area into the building. Although wild *Ae. aegypti* mosquitoes were found from the ground floor up to the top floor before releasing at Mentari Court [22] and other sites [32,33,34], the time it took for the invasion to be fully established differed to some extent for blocks and floors, which again could be a reflection of movement rates from outside the area. However, differences in the breeding sites available in different buildings may also be involved. Unfortunately, most of the breeding sites at Mentari Court are probably cryptic and reflect building defects where water can enter and/or damaged pipes. Perhaps these differ between floors and buildings, whereas the incidence of breeding in household containers is probably similar across the site.

Mentari Court was the first *Wolbachia*-infected *Ae. aegypti* release site in Malaysia and the first *w*AlbB field release site in the world. Appropriate laboratory backcrossing, fitness quality checks, and field release strategies were key parts of the factors for the successful establishment of the *Wolbachia*-infected mosquitoes in this study site [16], building on prior release experiences in Australia [26]. For four generations, the strain of *w*AlbB *Ae. aegypti* was backcrossed to a colony with field-collected *Ae. aegypti*. The backcrossed line was examined for fitness as well as insecticide resistance to pyrethroids, organophosphates, and fenitrothion [16]. Adult *Ae. aegypti* were released every three floors, concentrating on areas around the stairs and elevator. Each release location was fixed and released by the same staff member. The ovitraps used for monitoring were also fixed at the same position in corridors of the building, and externally at lamp posts, trees, beneath water tanks, close to prayer rooms, and beneath fire extinguishers. Despite the use of this standardised approach, it was not possible to avoid the external elements and activities that may have affected *Wolbachia* invasion and persistence, such as human mobility, spillover of insecticides from fogging of neighbouring outbreak sites, weather changes, and COVID-19 movement restriction orders during the lockdown.

## 5. Conclusions

In conclusion, the *Wolbachia* strain *w*AlbB was effectively established across the entire area covered by Mentari Court, and it has been persistent regardless of differences in buildings and across different floor levels. Although invasion within this area may have been indirectly influenced by *Ae. albopictus*, there is no evidence that this species has increased in incidence across the area. These results highlight the potential of *w*AlbB *Wolbachia* releases in similar high-rise developments where *Ae. aegypti* numbers are high. Invasion by *Wolbachia* will vary from location to location as evidenced by *w*AlbB releases in other areas [16] as well as in the varied success of *w*Mel releases [13], and it is important to understand the factors involved in these variable results which still tend to be unclear. Comprehensive investigations on spatiotemporal patterns such as those undertaken here can help guide upcoming field release plans and the development of models that can help predict local success. Relevant factors that need further consideration include effective community engagement and support, controlled fogging and mass insecticide spraying activities, good boundaries of highways and surrounding space to ensure separation from other residential buildings, defective building structures that provide mosquito habitats, high densities of resident mosquitoes, and favourable support from the local community to ensure consistent releases.

## Data Availability

Data are available at http://dx.doi.org/10.17632/sr2vppg24f.1 (accessed on 12 March 2023).
